# Psychology of Drunkenness

**Published:** 1909-07

**Authors:** Eloise Shepherd (Prosperine)

**Affiliations:** Houston, Texas


For Texas Medical Journal.

### PSYCHOLOGY OF DRUNKENNESS.

BY ELOISE SHEPHERD (PROSPERINE), HOUSTON. TEXAS.

Energy of every kind and in every thing is dual; or a union of masculine and feminine in activity. This union is spirit, and it is creative. God is the acknowledged creator of all things, and we are told He is male and female. We believe it would be a point conceded by all thinkers, that as either male or female God could not be a creator at all; hence, the fact or principle of sex is a basic one. All energy, then, is sexal—not sexual—in character; and this sexality is spirit. Creative love is sex-energy, then, in activity.

Let us unfold these propositions and see where they lead relative to the very pregnant question of prohibition now engaging the attention of all christendom, and of our own country in particular.

Alcohol is called "spirits"; psychologically and essentially a far greater truth than generally supposed. It truly is the spirit; the breath; the vital principle; the very life—whatever term best pleases wherewith to express the interior thought—distilled from fruit, grain or vegetable wherein the liquor-involved principle that intoxicates, is held; and which causes to appear on this visible plane, the forms of fruit, grain or vegetable so used. Examine a common bean or grain of corn. The bulk of the seed is merely its body; a body formed by its inner life in obedience to law, for clothing and protecting the chit; or visible expression of the inner life. The outer body of bean or corn may be assimilated by and so sustain the outer bodies of humanity; but the chit—in substance—alone feeds the real, the soul being, and affords it sustenance—speaking in a material sense of that which is immaterial—proof of which is found in the fact that with the chit removed from any seed it can not grow, and its body decays into dust. Prohibit these forms of life in the vegetable kingdom wherein their righteous work is being wrought, and all visibility, bereft of its sustaining forces, would vanish; would resolve again into formless matter. I repeat: all visibility would disappear, because the same law applies in the three kingdoms now known to man—mineral, vegetable and animal—with the spirit of the Creator dwelling in each. Man, the crown of animal creation, could no more escape the great devastation than any of the kingdoms below him. Rather would his destruction be more complete since his being is more dependent upon this inner spirit at which the man-made code of universal and arbitrary prohibition is aiming its death blows, and aiming them none the less fatally because aimed ignorantly. This creative spirit serves its holy and God-ordained purpose while operating unmolested within its heaven-appointed bounds; and in so fulfilling the law, exalts, sustains and makes perfect every objective form it impregns.

Let us carefully note before proceeding: It is not while following its righteous course and fulfilling its divine order that spirit is reversed or perverted; thus becoming a destructive instead of a creative or constructive force. We will bear in mind that force is here distinguished from power in that force is power in activity, in motion. Illustrating: We recognize steam as ice in activity. There could be no steam without the united elements constituting water; neither could there be ice. Both depend upon indestructible substances in combination, and all obedient to infinite law. Pervert this law and what, for want of a better sound-form, might be called "Intoxication, or Drunkenness of the Elements" would follow. Particles in ice-form vibrate at a low rate, while the same particles in steam-form vibrate many degrees higher. Why? Ah, the spirit; the life of fuel; i. e., coal or wood or even oil; extracted or liberated from materials wherein it had remained latent until freed by combustion, applied to ice, serves to raise the vibratory forces necessary for this transmuting of power in static ice to force in active steam. Spirit; liberated spirit, does this; see? Controlled and regulated by reason, reason guided by knowledge, and all ruled over by intelligence, or wisdom, only beneficent results accrue to the race so utilizing these wonderful forces. But turn them loose unreined, ungoverned, death and devastation marks their way, even as the tree riven by lightning or the blackened area swept by forest fires tell the story of destroying agencies holding sway over them.

As stated, every fruit, plant and vegetable grown contains this vitalizing substance known as spirit; remembering, also, that this spirit constitutes its life, and its life is its happiness, its living, its divine joy. What would drunkenness of the plant life be? Would not an undue portion, an excess of spirit or life—if it could be secured—cause the plant to lose its poise, its balance, its sobriety or sanity? Imbibing alcoholic fluids sufficiently, men lose control of mind and body, and we say "they are drunk." We have but to review the above stated facts, then observe that man's genius has devised methods whereby to extract, to distil this spirit from fruits and grains; and, under the delusion that an excess of such spirit taken into his system as drink, will make him happier, he proceeds to intoxicate his senses, lose his reason and degrade his whole being through the identical principle which the Creator uses to create, and exalt and make perfect every creature of His hand. Because a thing is good it doesn't follow a man should make a swinish glutton of himself, debauch his body, deface creation and shame the earth.

This spirit, this life, love, all good, lies about and throughout the planes of visible and invisible being; indeed, it constitutes being, itself, and can no more be exhausted by finite forms than the fishes of the sea can swallow all the water in which they swim. Mankind could inspire; could inbreathe; and, through meditation, prayer, temperance and a righteous observance of divine law so impregn himself with this infinite life principle as that he would have no need to distil it from fruit or grain, or prey upon another personality for it. It is his heritage as it is that of the lily or the rose; or of the fruit of tree or vine; and in this divine manner of indrawing his own, he would grow into the veritable likeness of his Creator. Instead, he distills the portion of spirit allotted to other objects; steals their life away, as it were; and so, overcharging his body with extracted forces, divine though they be, forces too intense in their vibrations for his physical being to endure, he is slain and blasted in his personality; and by the very substances which, righteously used, would have redeemed him from ignorance and death forever.

A gluttonous over-appropriation of "spirits" in any form or from any source, intoxicates—paralyzes—first the brain-organ, then extending throughout the entire nervous system; dethrones the reason, debauches the character and destroys the splendid form of man made in the image of God. Reason is humanity's balance wheel. Cast loose from its proper place in the organism, denied its righteous control of the individuality, and insanity to some extent follows. Why do men drink stimulants when weak, ill or troubled? Ah, they are weak, ill or troubled simply because of depleted spirit-force in their bodies. This spirit-force has been lost through dissipation or ignorance or some unspiritual cause, possibly a heritage from parents who sinned against them in the same manner while they were yet unborn; and the victim of either his own or another's sin does not know he could inhale, indraw, inbreathe straight from the inexhaustible fountain of creation all the supply he required did he but choose; nor resort to swallowing the "spirit-principle" of which some plant or grain has been robbed. Being weak or ill or troubled, he seeks three drinks, to ease physical pain, and to drown or forget anxiety and unrest of a physical mechanism that depleted spiritual power has failed to animate. The human instrumentality charged, yea, overcharged, with spirits mechanically drawn from the minor kingdoms, is soon clogged with abnormal appetites; and these demand more and more frequent indulgence. They lead to gluttonous revelry, and a drunken debauch the gratification of carnal desires.

Why the great prohibition movement? Why are so many people moved by a zealous desire to prohibit the sale and manufacture of spirituous beverages? Solely and alone because they lack understanding and want intelligence of laws governing the value and righteous use of this mighty life-agency; this great principle of Go(o)d; the spirit and Creator of universes, of worlds and of the inhabitants thereof. Education as to its use and proper regulation, and not its prohibition, is what the race today needs most direly.

Then there remains still another; a much more vital and far-reaching phase of this same question anent the use of intoxicants or spirituous liquors. It is a phase that conventionality frowns upon with severity whenever the slightest hint is given relative to its factorship in existence. It is the human creative impulse of the race; and wherever this letter shall be read, every man who reads it, though he may loudly dissent, will silently give assent. Men drink alcohol to promote their procreative forces. Let us consider the proposition from this viewpoint.

No truly enlightened member of the race, no one holding the best interests of humanity at heart, will deny that the gigantic system of commerce now based upon the brewing, distilling and extracting industries engaged in stealing from the vegetable kingdom this "more abundant life," then manufacturing and distributing it in the form of intoxicating liquors, is a very wrong use of a very righteous and beneficent principle. In the human animal, man, we see a curious compound of spirit, and, for want of a better term, anti-spirit, not found in any of the other natural kingdoms. They conflict; and they conflict because they are not acquainted; don't really know each other; much the same reason why nations go to war with one another. Man is finite, human, but his largest dowerage partakes of the infinite-divine; and the infinite-divine is spirit. Spirit being creative, man's strongest impulse is pro-spirit, or procreative. Remembering that spirit is always a supreme good, but that an excess of it in any sphere or form unfitted or unprepared for it, is, at the same time a dread evil, yet we step just here upon the real battle ground; the field of fiercest conflict between the carnal and the spiritual forces of the race. Unregenerate man derides the suggestion of striving after spirituality and its riches through soul development; claiming that, being a creature of the material earth, his greatest activities are properly in the sphere of the physical; overlooking the obdurate truth that these very so-called physical appetites and fleshly propensities are first, foremost and always of the spirit, hence spiritual in their innermost and outermost nature; must necessarily be so, else they could not be at all; since all that is, whether cognized as material or spiritual, is utterly dependent upon the great first cause, i. e., spirit, for being in any form or sense. Failing to cognize their spiritual character and deeming them "of the earth earthy," and therein to be utilized, he straightway perverts the force, thereby destroying his body through dethroning the very creative principle that built his physical being into existence; and so doing he degrades the ruler, the king of his own life, to the low plane of bodily sensuality. Still unseeing and unperceiving fully, yet craving a larger scope of power in the realm of creation, he seeks undue and excessive exercise of this sacred, this divine function, i. e., procreation. He finds the carnal and animal mechanism weaker than his desires, then strives to promote their strength through an assimilation of "spirits," drawn, not from divine to human springs, but from divine sources through human methods or channels; and his efforts along this specific line have been the chief factors engendering the monstrous whisky traffic, now deemed a menace—and rightly so deemed—to the progress and exaltation of the race. I repeat; I emphasize: the entire system of breweries and distilleries throughout the world today have for their basis, directly and indirectly, their chief cornerstone, the carnal appetites of man in his efforts to magnify in himself the human-creative, or procreative tides and forces. Man dimly senses the shining truth that spirit is good; and its use is also good; but he fails to grasp the infinitely greater truth of that godly Go(o)d which all mankind will eventually be required to aid in promoting; and in so promoting, promote true godliness in the earth, which is the real mission of the race.

Carnality—Lucifer—drew a fourth part of heaven away from the heavenly throne, thereby establishing hell, accepting the orthodox recital of the times. Sensuality is the fallen angel himself, personified here; and he is securing hosts of followers who willingly inscribe their names upon his banner. But shall all heaven be abolished or prohibited therefor? Because one certain dweller in holy spheres preferred the fires of the senses, and descended into the bottomless pit to enjoy them, leading thither all who were similarly inclined, is heaven less heaven and its glories less desirable to those who understand how to glorify and not destroy themselves with the righteous use of "spirits?"

Observe, now; spirit is spirit still; though having made and now remaining in, hell—if there be such a place—but it is spirit ill used, misplaced, and wrongly appropriated. What then? Stamp spirit out of existence, were such a thing possible? Abolish, prohibit, destroy the source and cause of all things? Absurd! How can the finite destroy the infinite? Rather teach these "fallen angels," these who are so ignorant of the place, use and worth of "spirit" or "spirits," even in drink. Teach them of its glory, its holiness, its redeeming power, and not of its destruction, its prohibition and annihilation; without which, as already stated, nothing of either a heavenly or a "helly" nature could remain; but that it—psirit—should be exalted, upborne and magnified for the recreation and rehabilitation of the spiritual in the humanal; and not for the degradation and utter damnation of the human by seeking its use merely for gratification of licentious appetites.

Spirit is always divine, either in or out of alcoholic beverages, and should be assimilated for the reflection of divinity in humanity. Once the individual understands that in so far as he secures additional draughts of Go(o)d or spirit from every legitimate source, then utilizes it for promoting his spiritual strength, strength conserved for use in spiritual spheres, and not for dissipation in drunken debauchery, then, and not until then, will prohibition, in so far as it should be such, prohibit; and then it will prohibit without regulation through civil codes or enactments for enforcing what the enlightened understanding prefers accepting voluntarily rather than being forced by law to observe.

The world's writers and teachers cry aloud over the intoxication of the race through the use of alcoholic beverages, or "spirits," and they should so cry; but this last named form of intoxication is smiting humanity with a far deadlier stroke, as all may see who look. Forms of mankind are walking the earth, blasted and withered, and shorn of graces both physical and intellectual; and more through intoxicated procreative forces than any other cause. Is this statement met with dissent? See the dread spectacle of overcrowded prisons and insane asylums defacing the entire land; with directors and superintendents constantly calling on the State for more room; accommodation for more victims. Read the statistical reports of these prison and asylum guardians and note the awful increase of patients and criminals flowing from all points for restraint of some kind. Cause? Cause there must be, else no effects would follow. Wherever a family has a member falling a victim to insanity they are almost as disposed to conceal the fact as though he had been guilty of crime. Why? A sense of shame or disgrace attaches to the disease known as dethroned reason. Deep within the souls of men they know they violated a heavenly command when they debased the divine to human planes through abuse of creative privileges. Men lose their reason temporarily through drinking intoxicating liquors; but, alas, and alas! they often lose it forever from a false impression or idea that they are gaining enjoyment in the realm of sensualism. Descending to a plane of utterly dissipating the divinest forces extant in nature, wasting their very life and draining their highest vitality in the form of sex-intoxication, as surely destroys the body with the disease called insanity—softening of the brain—as it is frequently termed for courtesy's sake, as that the lightning can blast the splendid tree but erstwhile waving its branches in the happy breezes, expressive of its delights in being.

The tiniest insect can burrow, little by little, into the strongest timbers of the finest structure that ablest and wisest architects can rear, in time undermining and destroying the whole achievement. And, even so, the subtlest dissipations, debaucheries and indulgencies, practiced or imposed, can and will as surely commit to ruin the utmost perfection of the individuality; bodily, mentally and spiritually. The insect enjoys the burrowing. Assuredly; and did it but know the truth, then govern its course accordingly, nature has furnished perfect conditions for the exercise of its native propensities; but they exist where no harm could befall higher works. In the insect's proper sphere, it may, as in the case of coral, commute a baser into a finer substance, thus aiding the process of evolution; but when its energies are engaged in tearing down what a wiser intelligence has constructed, it becomes destructive instead of constructive.

Individually, members of the race have but to seek somewhat of wisdom along these lines, than apply it to their personal existence; and the fruitage of such application will declare whether they are "serving God or Mammon."

